# Hepatic Rupture as the Initial Presentation of an *EGFR*-Mutated Lung Adenocarcinoma: A Case Report

**DOI:** 10.3389/fonc.2022.837630

**Published:** 2022-03-30

**Authors:** Oriol Mirallas, Marc Bosch-Schips, Nuria Pardo, Anton Aubanell, Maria Teresa Salcedo-Allende, Ana Callejo, Patricia Iranzo, Josep Tabernero, Enriqueta Felip

**Affiliations:** ^1^ Medical Oncology Department, Vall d’Hebron Hospital Campus and Vall d’Hebron Institute of Oncology (VHIO), Barcelona, Spain; ^2^ Hematology Department, Vall d’Hebron Hospital Campus and Vall d’Hebron Institute of Oncology (VHIO), Barcelona, Spain; ^3^ Radiology Department, Vall d’Hebron Hospital Campus, Barcelona, Spain; ^4^ Pathology Department, Vall d’Hebron Hospital Campus, Barcelona, Spain

**Keywords:** non-small cell lung carcinoma, *EGFR* mutations, metastatic hepatic rupture, tyrosine kinase inhibitor, case report

## Abstract

Hepatic rupture is a rare complication of solid tumor malignancies, notably in lung adenocarcinomas, and carries an extremely poor overall prognosis. Epidermal growth factor receptor (EGFR) mutations in lung adenocarcinoma predict benefit with tyrosine kinase inhibitors (TKIs). This case report describes a female patient who presented with a metastatic hepatic rupture and was subsequently diagnosed with *EGFR*-mutated lung adenocarcinoma. The tumor had an impressive response to TKI inhibitor treatment, reversing her extremely poor, short-term prognosis. We believe this unique case sheds light on the treatment management of hepatic ruptures and supports the high response rate seen with TKIs in *EGFR*-mutated lung cancers, regardless of the patient’s performance status.

## Introduction

The discovery of epidermal growth factor receptor (*EGFR)* mutations has revolutionized the treatment of non-small cell lung cancer (NSCLC). Approximately one third of NSCLC harbor an EGFR mutation, being more likely in Asian, female, and non-smoker patients with adenocarcinoma histology (1). First and second generation EGFR tyrosine kinase inhibitors (TKIs) have reported outstanding response rates, ranging from 60% to 80% in the IPASS, OPTIMAL, EURTAC and LUX-Lung 7 phase 3 clinical trials (2–5). In the case of third generation TKI osimertinib in first-line setting, benefit over gefitinib/erlotinib was demonstrated in regard to median progression-free survival and overall survival in the FLAURA trial. *EGFR* mutation-positive patients with poor ECOG PS 3-4 benefit from EGFR-TKIs (6). The current standard first-line therapy for patients with metastatic NSCLC harboring an activating *EGFR* mutation is an EGFR-directed oral TKI (7).

Whilst hepatic rupture in previously diagnosed liver metastasis has been reported, it is usually associated with traumatic events, and presentation as the initial manifestation of liver metastasis or tumor diagnosis is an uncommon occurrence. It is most commonly seen in primary liver hepatocarcinoma (8), whereas to our knowledge, there are only two published accounts of lung adenocarcinoma as a cause of hepatic rupture, although they have been reported more frequently with other lung cancer types, especially small cell lung cancer. Of note, both of these reported lung adenocarcinoma cases had an unhealthy liver; one patient was positive for the hepatitis B virus and the other had extensive alcohol consumption (9, 10). None of the cases described reported the molecular status of the lung neoplasm. Computed tomography angiogram (angio-CT) is the preferred diagnostic method for ruptured neoplastic liver disease, and contrast-enhanced ultrasonography is useful as a road-map for angiography. In many cases an active bleeding target could be identified, and in this situation embolization procedures were preferred. On the other hand, when an active bleeding point cannot be identified, packing and supportive care are the preferred treatment options, with trans-arterial embolization being considered when conservative measures are insufficient (11). Regardless of the treatment administered, hepatic rupture has a >50% in-hospital mortality and more than 90% of patients die within the first 6 months after discharge (11).

We report a case of an *EGFR*-mutated lung adenocarcinoma due to the exceptionality of a hepatic rupture as the initial presentation in a healthy liver of a young woman and her subsequent spectacular tumor response to TKI inhibitor treatment.

## Case Presentation

A 53-year-old Caucasian woman with no toxic habits and no relevant personal or family health history was admitted at our hospital due to abdominal pain that had worsened over the last two days. At admission, she presented with an ECOG performance status (PS) of 3 and described a monosymptomatic generalized abdominal pain. Physical examination revealed the patient was pale, tachycardic, tachypneic, poorly perfused, and had normal blood pressure, with no signs of exteriorized active bleeding. Abdominal palpation showed tenderness predominantly in the right hypochondrium. Initial venous blood gas analysis showed a hemoglobin level of 7.2 g/dL with a pH of 7.35 and a lactate elevation of 3.7 mmol/L. Blood tests showed hemoglobin of 6.3 g/dL, WBC count 23.19x10^9^/L, creatinine 1.51 mg/dL, hepatic cytolysis with aspartate aminotransferase of 722 UI/L and alanine aminotransferase of 1114 UI/L, alkaline phosphatase of 476 UI/L, gamma-glutamyl transferase of 418 UI/L, bilirubin within range, an absence of altered coagulation parameters, and C-reactive protein of 5.37mg/dL. Blood cultures were performed, and SARS-CoV-2 PCR was negative.

**Figure 1:**
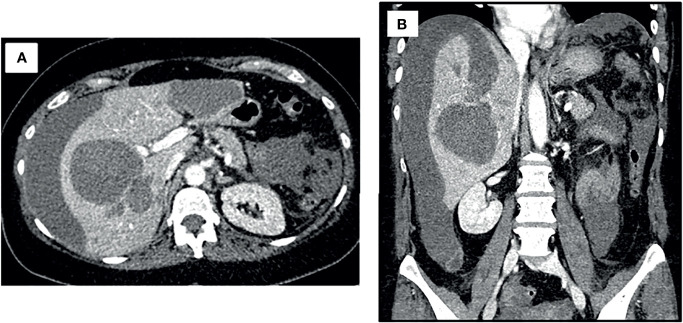
Angio-CT at initial case presentation. Both axial **(A)** and coronal **(B)** views revealed two hepatic cystic lesions; one in each hepatic lobule with signs of bleeding at different coagulation stages. The left cystic lesion opened into the hepatic subcapsular space producing a hemoperitoneum, without signs of active bleeding.

Upon suspicion of hemorrhagic shock, crystalloid fluid resuscitation, broad spectrum antibiotics and packed red blood cells were administered, achieving hemodynamic stability. To identify the cause of hemorrhage, an angio-CT was performed, revealing two hepatic cystic lesions, one in each hepatic lobe with signs of bleeding at different coagulation stages. The left lesion opened into the hepatic subcapsular space producing a hemoperitoneum, without signs of active bleeding (**Figure 1**).

A conservative approach was chosen due to the patient’s stability and no demonstrable active bleeding. The patient was hospitalized in the semi-critical unit, where she remained hemodynamically stable, albeit with progressively worsening anemia which required transfusion of two packed blood cells daily. Upon worsening of her condition and the addition of an abdominal compartmental syndrome, a new CT scan showed cholangitis with hepatic phlegmonous repercussion of the II segment, without an active bleeding site susceptible to embolization. A surgical exploratory laparoscopy was performed, finding four liters of blood in the abdominal cavity, a ruptured hepatic low output bleeding capsule laceration in the antero-medial region and a congestive liver with two heterogenous tumors. Intraoperative biopsies were taken and revealed a multifocal malignant carcinoma, and a hepatic resection was therefore dismissed. Exhaustive peritoneal washing was performed, and hemostatic intra-laceration agents were applied.

**Figure 2:**
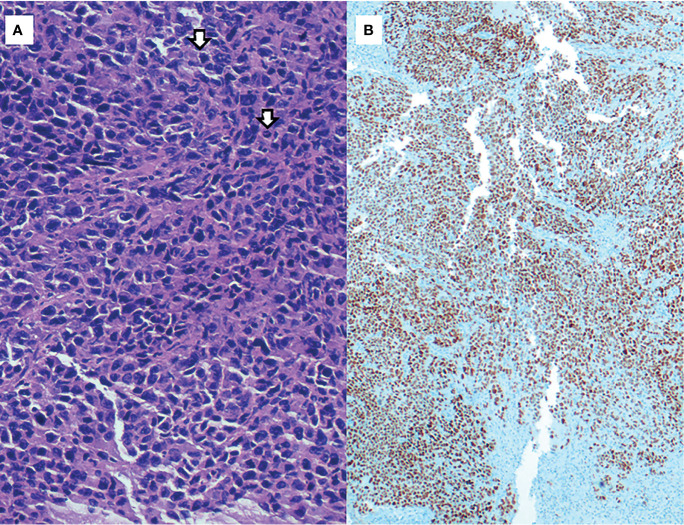
**(A)** H&E, 400x: High-grade adenocarcinoma, with a solid growth pattern and mitotic figures (arrows). **(B)** Immunohistochemical staining of TTF-1 (200x): strong and diffuse nuclear positivity of neoplastic cells.

**Figure 3:**
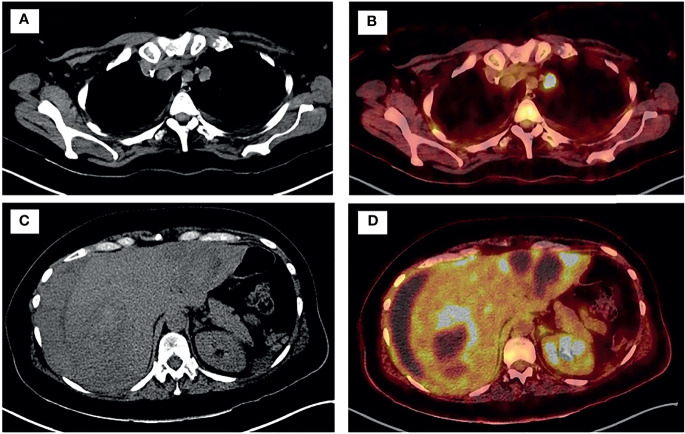
PET-TC showed disseminated disease in both lungs **(A, B)**, supradiaphragmatic adenopathy, tail of the pancreas, multiple bi-lobar hepatic focal areas **(C, D)** and in several vertebrae.

After surgery, the patient remained hemodynamically stable, but remained dependent on multiple blood transfusions (two packs approximately every two days). Pathology revealed the presence of hepatic infiltration due to a high-grade carcinoma, with a solid growth pattern, areas of necrosis and mitotic figures. This led to an intense and diffuse nuclear positive immunohistochemical study for TTF-1 and a focal cytoplasmic analysis for cytokeratin cocktail, which was negative for neuroendocrine markers. Together, the findings were compatible with liver metastasis from high-grade adenocarcinoma of lung origin (**Figure 2**). The molecular study showed negativity for *ALK* and *ROS-1* translocations, and low PD-1 (<1%) expression. Polymerase chain reaction (PCR-COBAS EGFR Mutation Test) screening for *EGFR* mutations showed an exon 19 deletion. Hence, a primary lung adenocarcinoma was suspected, with a CT scan showing a 22 x 20 mm para-mediastinal nodule in the superior lobe of the left lung with adenopathic involvement of the mediastinum (**Figures 3A, B**). Positron emission tomography showed dissemination in both lungs, a supradiaphragmatic adenopathy, multiple hepatic focal areas, as well as involvement in the pancreas tail and several vertebrae (**Figures 3C, D**). A brain CT scan was normal.

Our 53-year-old female patient was diagnosed with a stage IVc exon 19 mutated *EGFR* lung adenocarcinoma with a high burden of disease, which presented as a metastatic liver rupture with hemoperitoneum. EGFR-TKI treatment was opted for, since only Erlotinib was readily available. Third generation EGFR-TKIs needed long approval procedures at our center, which would have delayed treatment initiation and risked the patient’s wellbeing. Therefore, Erlotinib was started at a dose of 150 mg/daily. Her blood transfusion needs rapidly decreased, with stabilization of hemoglobin levels after 72 hours of TKI treatment. This clinical symptomatic response was further supported by an angio-CT which showed a rapid decrease of the hemorrhagic component and subcapsular hematoma, and closing of the capsule laceration, with minimum remaining hemoperitoneum. A summary of the case evolution can be found in **Table 1**.

**Table 1:** Treatment timeline.

Days		CASE EVOLUTION
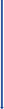	**0**	Hemorrhagic shock and ECOG PS* 3
	**0**	Hepatic rupture diagnosis
	**2**	Anemia which required transfusion of two packed blood cells daily.
	**4**	Exploratory laparoscopy and packing
	**4**	Anemia which required transfusion of one to two packed blood cells daily.
	**6**	Diagnosis of exon 19 deletion of *EGFR* and initiation of erlotinib treatment
	**9**	Clinically stable and no more transfusions
	**90**	ECOG PS 0 and a partial response (-55%) in the first CT scan evaluation.
	**180**	Maintained partial response with excellence tolerance.

*PS, performance status.

**Figure 4:**
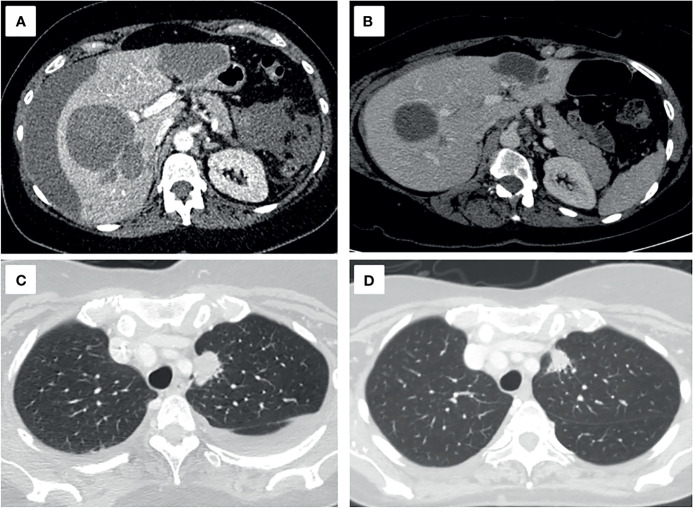
Axial projections of hepatic **(A, B)** and pulmonary **(C, D)** target lesions, in the diagnostic **(A, C)** and follow-up CT scan three months later **(B, D)**. A decrease of 55.1% of the sum of the longest diameters, a partial response, based on the RECIST 1.1 criteria is seen.

The patient was discharged after a one-month hospitalization, with an ECOG PS 1. Follow-up after 3 months showed an ECOG PS 0 and a partial response with a 55% decrease in tumor volume (**Figure 4**).

## Discussion and Conclusion

Hepatic rupture is a rare complication in patients with solid tumor malignancies, especially for lung adenocarcinomas, and carries an extremely poor prognosis. Remarkably, no *EGFR* mutated cases have been reported and at the time of this report, no standardized treatment has been established.

Our patient partially improved after applying local hemostatic agents, but it was not until the targeted TKI therapy was initiated that the hepatic hemorrhage resolved with no further need for transfusions. The high overall response rates reported in clinical trials with erlotinib, as well as TKI’s short time-to-partial response, could explain the bleeding halt that could not be achieved *via* local hemostatic methods alone (3, 12), Although the initial patient outcome was deemed poor, surgical management in combination with a rapid hemorrhagic tumor response resulted in an excellent outcome, including a partial response in the follow-up CT scan three months later. In these very particular cases, systemic medical therapies should be considered, regardless of the patient’s PS. We believe this unique case sheds light on the treatment of hepatic ruptures, especially in targetable tumors.

## Patient’s Perspective

I was hospitalized full of fear and uncertainty. Physicians did not know where the bleeding came from, and my prognosis was very poor. I was prepared to die, but I could not believe my day was coming so fast. After the molecular results came out, oncologists told me about a possible targeted treatment, and I still cannot believe the rapid improvement I experienced. In less than four days I was walking in the room without any blood transfusion and in a week, I was able to come back home. This targeted treatment saved my life.

## Data Availability Statement

The original contributions presented in the study are included in the article. Further inquiries can be directed to the corresponding author.

## Ethics Statement

Written informed consent was obtained from the individual for the publication of images or data included in this article.

## Author Contributions

OM and MB contributed to the conceptualization, methodology, investigation, and resources and writing of the original draft. NP contributed to the conceptualization, investigation, and writing review and editing of the manuscript. AA did the data curation and obtained the CT scan images. MS-A obtained the pathological images and description and helped with data curation. AC helped with the validation, investigation, and writing review and editing of the manuscript. PI performed the formal analysis and writing review and editing. JT supervised and helped with the funding acquisition. EF supervised and validated the final manuscript and helped with the funding acquisition. All authors contributed to the article and approved the submitted version.

## Conflict of Interest

The authors declare that the research was conducted in the absence of any commercial or financial relationships that could be construed as a potential conflict of interest.

## Publisher’s Note

All claims expressed in this article are solely those of the authors and do not necessarily represent those of their affiliated organizations, or those of the publisher, the editors and the reviewers. Any product that may be evaluated in this article, or claim that may be made by its manufacturer, is not guaranteed or endorsed by the publisher.
